# Association between fibrinogen and decline in cardiorespiratory fitness in patients undergoing cardiopulmonary exercise testing: a large cross-sectional study

**DOI:** 10.3389/fcvm.2026.1737141

**Published:** 2026-03-24

**Authors:** Jiayuan Zhang, Hongxin Dong, Shengyi Lei, Yan Lu

**Affiliations:** 1 Department of Cardiology, First Hospital of Shanxi Medical University, Taiyuan, Shanxi, China; 2 School of Computer Science and Technology, North University of China, Taiyuan, Shanxi, China; 3 School of Public Health, Shanxi Medical University, Taiyuan, Shanxi, China

**Keywords:** biomarkers, cardiopulmonary exercise testing, cardiorespiratory fitness, fibrinogen, subgroup analysis

## Abstract

**Background:**

Cardiorespiratory fitness (CRF) is critical cardiovascular risk predictor but unavailable for routine screening. Fibrinogen (FIB), a widely available biomarker, may be related to CRF through multiple mechanisms. However, evidence linking FIB to CRF decline is unclear. Therefore, we aimed to evaluate the relationship between FIB and CRF decline and to further examine its association with peak O_2_ pulse to provide physiological insight.

**Methods:**

In this cross-sectional, retrospective study, 698 participants were included. FIB levels were categorized into tertiles: T1 (*n* = 238), T2 (*n* = 228), and T3 (*n* = 232). CRF status was divided into two groups based on CPET: normal-to-mild decline (*n* = 292) and moderate-to-severe decline (*n* = 406). Univariate and multivariate logistic regression, subgroup, sensitivity, and receiver operating characteristic (ROC) analyses were performed to evaluate the relationship between FIB and CRF decline. Multivariable linear regression was performed to examine the association between FIB and peak oxygen pulse (peak O_2_ pulse).

**Results:**

The prevalence of moderate-to-severe CRF decline increased across FIB tertiles (T1: 49.6%, T2: 60.1%, T3: 65.1%; *P* = 0.002). In the fully adjusted model of multivariate logistic regression, each 1-unit increase in FIB was associated with a 31.4% higher risk of moderate-to-severe CRF decline (OR = 1.314, 95% CI: 1.051–1.643, *P* = 0.016). Participants in theT3 had a 1.620-fold higher risk compared with those in T1 (OR = 1.620, 95% CI: 1.101–2.383, *P* = 0.014). The association remained consistent across all subgroups and was stronger in sensitivity analyses restricted to participants achieving maximal effort (*P* < 0.05). ROC analysis revealed a predictive value of FIB for moderate-to-severe CRF decline (AUC = 0.584, *P* < 0.001). In fully adjusted linear regression, each 1 g/L increase in FIB was associated with a 0.357 mL/beat lower peak O_2_ pulse (*β* = −0.357, *P* < 0.001), and T3 had a lower peak O_2_ pulse than T1 (*β* = −0.503, *P* = 0.012).

**Conclusions:**

Among individuals undergoing CPET, higher FIB levels were associated with higher risk of moderate-to-severe CRF decline and lower peak O_2_ pulse, suggesting that FIB may serve as a potential biomarker for identifying CRF decline.

## 1 Introduction

Cardiovascular disease (CVD) remains one of the leading causes of death worldwide. Between 1990 and 2019, CVD-related deaths increased from 12.1 million to 18.6 million (1). Projections suggest that by 2050, the global prevalence of CVD will rise by approximately 90%. Crude mortality is expected to increase by 73.4%, reaching an estimated 35.6 million cardiovascular deaths (2). The growing public health burden of CVD underscores the urgent need for precise risk assessment and early intervention. Importantly, clinical practice must not only focus on patients with established CVD but also extend to individuals presenting with cardiac-related symptoms (e.g., chest pain, unexplained dyspnea, reduced exercise tolerance) who have not yet been diagnosed with structural heart disease. These individuals often represent a high-risk population; therefore, early identification of functional impairment and reduced cardiopulmonary reserve is particularly important (3–5).

Cardiorespiratory fitness (CRF) reflects the integrated capacity of the respiratory, cardiovascular, and muscular systems to take up, transport, and utilize oxygen (6). Substantial evidence has shown that CRF provides superior or complementary prognostic value compared with traditional risk factors in predicting cardiovascular outcomes and all-cause mortality (7, 8). Meta-analyses consistently show a dose–response relationship between CRF and outcomes: each 1-MET higher CRF is associated with an approximately 11%–17% lower risk of all-cause mortality and fewer CVD events (7). This association appears largely independent of body mass index (BMI) (8). Currently, the gold standard for CRF assessment is cardiopulmonary exercise testing (CPET), which evaluates multiple parameters such as peak oxygen consumption (VO_2_ peak) and peak oxygen pulse (O_2_ pulse) (9). However, the widespread application of CPET for population-level screening and primary care practice remains limited due to constraints of accessibility, cost, and time (10). Therefore, identifying and validating routinely available clinical biomarkers as surrogate indicators for rapid screening, risk stratification, and predictive modeling could complement CPET, enhance risk detection efficiency, and optimize healthcare resource allocation.

From a pathophysiological perspective, potential biomarker candidates should be linked to mechanisms that influence oxygen delivery and utilization during exercise. Accumulating evidence from epidemiological and clinical studies indicates that inflammation and coagulation-related biomarkers are not only closely associated with adverse cardiovascular outcomes but may also influence CRF (11–13). When selecting biomarkers for real-world screening and risk stratification, we prioritized indicators that are routinely available and biologically plausible. Cytokine- or fibrinolysis-regulatory markers (e.g., IL-6, TNF-α, and PAI-1) are not routinely measured and are susceptible to assay-related variability, limiting their comparability across settings (14–16). Although high-sensitivity C-reactive protein (hs-CRP) and D-dimer are more commonly used, they frequently fluctuate with infection/inflammation or tissue injury; notably, D-dimer specificity decreases with age, increasing the rate of false positives (17–19). In contrast, fibrinogen (FIB) demonstrates more robust clinical interpretability. Recent evidence suggests that conventional standalone coagulation parameters often fail to capture mechanistically relevant abnormal phenotypes, such as “subclinical hypercoagulability”. In this context, a decline in fibrinolytic potential driven primarily by FIB has been identified as a mechanistic pathway relatively independent of systemic inflammation, directly mediating the transition from metabolic derangement to prothrombotic state (20, 21). From a structure-function perspective, FIB is highly susceptible to oxidative stress-induced biochemical modifications within inflammatory environments. Such alterations significantly impact the properties of the resulting fibrin network, which collectively dictate the thrombotic phenotype (22). Consequently, FIB abnormalities are not merely byproducts of inflammation; rather, when mediated by oxidative stress, they may directly impair microcirculatory perfusion and oxygen transport by altering clot architecture and fibrinolytic balance (23). Concurrent innovations in detection technologies, such as surface plasmon resonance (SPR)-based biosensors, have enabled high-sensitivity quantification of FIB within clinical ranges (24). Together, these findings indicate that FIB is not only a well-established, cost-effective biomarker but also a pivotal pathological link bridging systemic inflammation, oxidative stress, and microcirculatory dysfunction. This provides a compelling mechanistic framework for explaining the association between impaired oxygen delivery/utilization and CRF decline. Consistent with this biological relevance, numerous studies have demonstrated that elevated FIB levels are associated with an increased risk of mortality from cardio-cerebrovascular diseases (25, 26). However, while the association between FIB and elevated cardiovascular risk is well-recognized, its relationship with CRF—particularly the dynamic functional parameters measured by CPET—remains under-explored.

To address this gap, the present study utilized a large, real-world clinical cohort to systematically evaluate the independent association between serum FIB levels and CPET-derived CRF. Furthermore, we investigated the association between FIB and peak O_2_ pulse. As a comprehensive surrogate for stroke volume and peripheral oxygen extraction, the inclusion of peak O_2_ pulse allows for a further elucidation of the potential physiological pathways through which FIB influences cardiorespiratory reserve. We hypothesized that elevated FIB is a sensitive indicator of CRF decline. By validating this association, we aim to provide a cost-effective and accessible clinical tool to assist in identifying high-risk individuals with functional impairment, thereby offering a robust evidence-based framework for precision risk stratification and early intervention.

## 2 Methods

### 2.1 Study population

**Figure 1:**
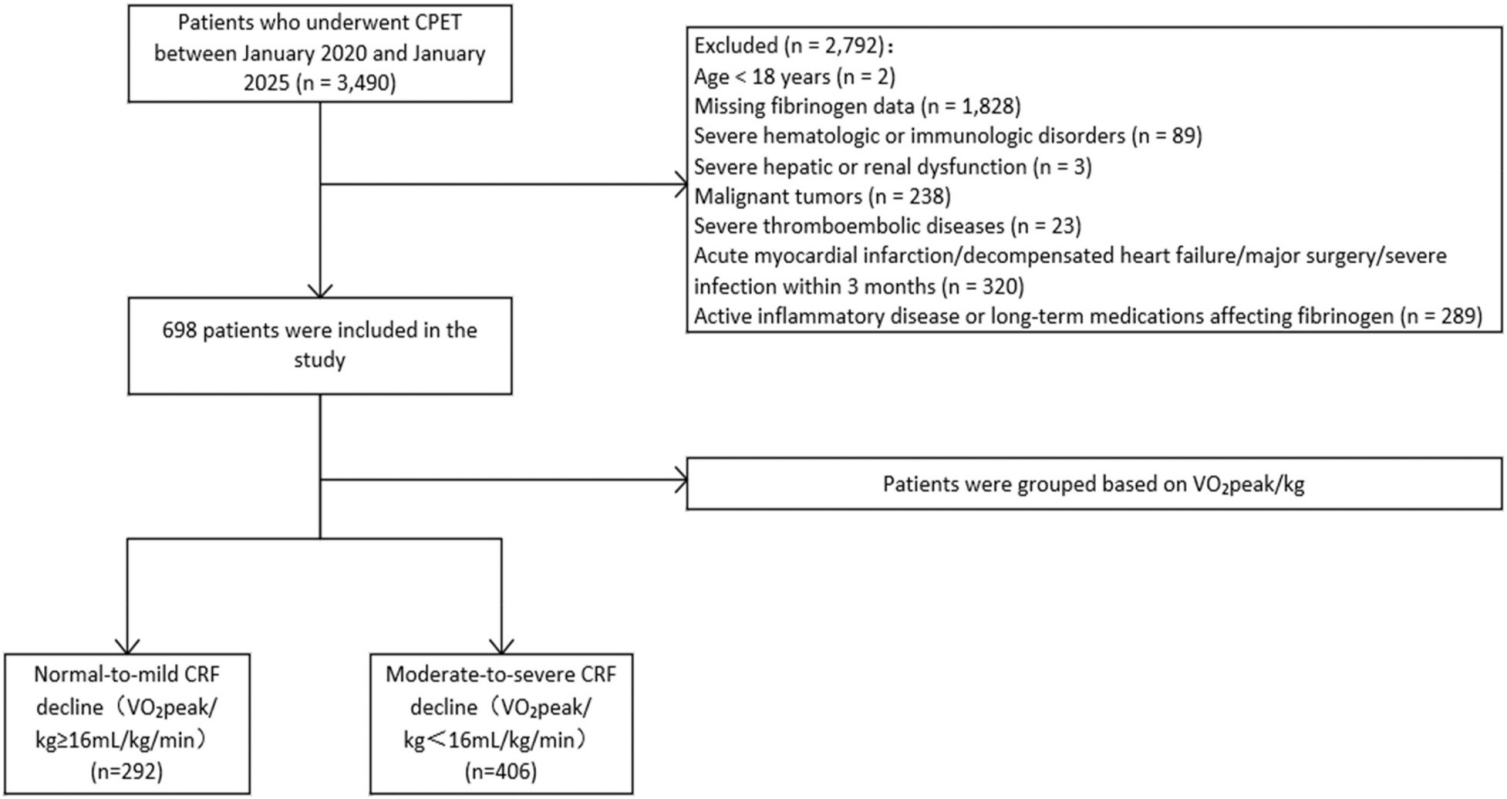
The ﬂowchart of patient selection. CPET, cardiopulmonary exercise testing; CRF, cardiorespiratory fitness.

This single-center, cross-sectional, retrospective study screened patients who underwent CPET at the First Hospital of Shanxi Medical University between January 2020 and January 2025. Inclusion criteria: (1) age ≥ 18 years; (2) completion of CPET during hospitalization. Exclusion criteria: (1) missing FIB data; (2) severe hematologic or immunologic disorders; (3) severe hepatic or renal dysfunction; (4) malignant tumors; (5) severe thromboembolic diseases; (6) acute myocardial infarction, decompensated heart failure, major surgery, or severe infection within the past 3 months; and (7) active inflammatory diseases or long-term use of medications significantly affecting FIB levels (e.g., anticoagulants, immunosuppressants, or high-dose glucocorticoids). After rigorous screening and application of the exclusion criteria, a total of 698 participants were ultimately enrolled in the final analysis (Figure 1).

The study protocol was approved by the Ethics Committee of the First Hospital of Shanxi Medical University on March 2, 2023 (No. KYYJ-2023-092) and was conducted in accordance with the principles of the Declaration of Helsinki. Given the retrospective observational nature of the study involving anonymized data and no interventions, the Ethics Committee waived the requirement for informed consent.

### 2.2 Data collection and definitions

Clinical data were retrieved from the hospital electronic medical record system. These data included demographic characteristics, anthropometric measurements, medical history and medication use, laboratory biomarkers, and echocardiographic parameters. Demographic characteristics comprised age, gender, smoking status, drinking status, and family history of CHD. Anthropometric measurements included height, weight, BMI, systolic blood pressure (SBP), diastolic blood pressure (DBP), and resting heart rate (HR).

Medical history included hypertension, hyperlipidemia, diabetes, stroke, CHD, history of percutaneous coronary intervention (PCI), and arrhythmias. Hypertension was defined as SBP ≥140 mmHg and/or DBP ≥90 mmHg on three separate measurements without antihypertensive treatment, a prior diagnosis of hypertension, or current use of antihypertensive drugs (27). Hyperlipidemia was defined as total cholesterol (TC) ≥ 6.2 mmol/L, low-density lipoprotein cholesterol (LDL-C) ≥ 4.1 mmol/L, triglycerides ≥2.3 mmol/L, a prior diagnosis, or current use of lipid-lowering medication (28). Diabetes was defined as fasting glucose ≥7.0 mmol/L, 2-hour post-Oral Glucose Tolerance Test glucose ≥11.1 mmol/L, glycated hemoglobin ≥6.5%, prior diagnosis, or current use of antidiabetic drugs (29). Stroke was defined as focal or global neurological deficits caused by cerebralvascular occlusion or rupture, lasting ≥24 h or resulting in death, excluding non-vascular causes (30). Medication use included antihypertensive, antidiabetic, lipid-lowering, antiplatelet and β-blockers therapies. Laboratory biomarkers included alanine aminotransferase (ALT), aspartate aminotransferase (AST), albumin, fasting glucose, TC, triglycerides, LDL-C, high-density lipoprotein cholesterol (HDL-C), estimated glomerular filtration rate (eGFR), uric acid, electrolytes (potassium, sodium, chloride), hemoglobin, white blood cell count (WBC), platelet count, D-dimer, and FIB. Left ventricular ejection fraction (LVEF) was obtained via echocardiography and expressed as a percentage.

### 2.3 Measurement and grouping of fibrinogen

FIB levels were measured using a fully automated coagulation analyzer (CS-5100; Sysmex, Japan) with corresponding reagents. For the analysis, FIB was analyzed both as a continuous variable and a categorical variable, the latter being divided into tertiles based on its distribution: T1 (≤2.58 g/L, *n* = 238), T2 (2.59–3.07 g/L, *n* = 228), and T3 (>3.07 g/L, *n* = 232). For each participant, FIB was obtained from the first measurement on admission, and a single FIB value per participant was used for analysis.

### 2.4 Cardiopulmonary exercise testing and cardiorespiratory fitness assessment

All participants underwent a single symptom-limited CPET using an advanced cardiopulmonary exercise testing system (CARDIOVIT CS-200; SCHILLER AG, Switzerland) under standardized conditions. The test was performed using a ramp protocol with progressively increasing workload until symptom limitation or attainment of standard termination criteria (31). During the test, breath-by-breath measurements of VO_2_, carbon dioxide output (VCO_2_), minute ventilation (VE), and respiratory exchange ratio (RER) were obtained using a metabolic measurement system. Data were averaged over 10 s intervals. Simultaneous 12-lead electrocardiography, heart rate, and blood pressure were continuously monitored. The anaerobic threshold (AT) was determined using the V-slope method (32), and ventilatory efficiency was assessed by the VE/VCO_2_ slope (33).

It is generally accepted that during a CPET, a VO_2_ plateau (or a decline) despite increasing workload is considered evidence of maximal effort. If no plateau was observed, the test was considered near-maximal when any of the following criteria were met: RER >1.10, breathing reserve (BR) < 15%, peak heart rate >90% predicted, or peak blood lactate ≥8 mmol/L (if available) (34). The termination criteria for the test include, but are not limited to: severe chest pain, significant ST-segment depression or elevation (≥2 mm), complex ventricular arrhythmias, excessive blood pressure elevation (SBP >250 mmHg or DBP >120 mmHg), marked hypoxemia (SpO_2_ < 80%), dizziness or syncope, or voluntary request by the participant to stop the test (35). All tests were supervised throughout by experienced physicians and technicians, with comprehensive emergency equipment available on site to ensure participant safety.

CPET provides multidimensional parameters that reflect cardiopulmonary, circulatory, and metabolic function. The primary outcome variable in this study was CRF, which was evaluated by measuring maximal oxygen uptake (VO_2_ max) or VO_2_ peak during CPET (36). In clinical practice, there is currently no unified CRF grading standard for the general or non–heart failure population. VO_2_ peak comprehensively reflects the reserve capacity of the cardiopulmonary system and exercise tolerance. Therefore, we referenced the functional classification system proposed by Weber et al., based on VO_2_ peak/kg (Class A > 20, Class B 16–20, Class C 10–15, Class D < 10 mL/kg/min) (37). This classification was originally developed to stratify exercise capacity in patients with chronic heart failure (38). Although the Weber classification was initially derived from the heart failure population, its physiological basis is also applicable to non–heart failure individuals. Subsequent studies and expert consensus have recognized its value as a reference framework for interpreting CPET results (39, 40), and a cutoff value of 16 mL/kg/min has been adopted in several studies to define impaired CRF (41). Based on this rationale and the characteristics of our study population, CRF was categorized into two levels: normal-to-mild CRF decline (VO_2_ peak/kg ≥ 16 mL/kg/min, *n* = 292) and moderate-to-severe CRF decline (VO_2_ peak/kg < 16 mL/kg/min, *n* = 406). This cutoff was applied to reflect the participants' overall CRF status rather than to perform disease-specific prognostic stratification. In addition, we performed an additional analysis of peak O_2_ pulse derived from CPET. Calculated as the ratio of VO_2_ peak to HR peak, peak O_2_ pulse represents the amount of oxygen consumed per heartbeat. Physiologically, it serves as a comprehensive surrogate for the product of stroke volume and the peripheral arteriovenous oxygen difference, thereby reflecting the integrated efficiency of central hemodynamics and peripheral oxygen extraction during maximal exercise.

### 2.5 Statistical analysis

All statistical analyses were performed using SPSS software (version 27.0; IBM Corp, Armonk, NY, USA). Continuous variables were first assessed for normality using the Shapiro–Wilk test and were found to be non-normally distributed. These variables are therefore presented as medians with interquartile ranges (IQR), and between-group differences were analyzed using the Mann–Whitney *U*-test or the Kruskal–Wallis H test. Categorical variables are presented as counts and percentages, and between-group differences were analyzed using the chi-square (*χ*^2^) test. To evaluate the association between FIB and moderate-to-severe decline in CRF, univariate logistic regression analysis was first performed to screen for candidate variables (*P* < 0.05). Subsequently, three multivariable logistic regression models were constructed: Model 1 was unadjusted; Model 2 adjusted for gender, drinking, and CHD; and Model 3 further adjusted for all potential confounders, including gender, drinking, CHD, fasting glucose, HDL-C, eGFR, hemoglobin, and LVEF. Subgroup analyses were conducted stratified by age, gender, BMI, smoking status, hypertension, hyperlipidemia, diabetes, and CHD to assess the robustness of the results. For the sensitivity analysis, multivariable analyses were repeated exclusively in participants who reached or approached maximal exercise effort (RER ≥ 1.10). In addition, multivariable linear regression was performed to employed the association between FIB and peak O_2_ pulse. Model 1 was unadjusted. Model 2 adjusted for gender, smoking, drinking and β-blocker use. Model 3 adjusted for gender, smoking, drinking, β-blocker use, age, BMI, DBP, ALT, platelet count, hemoglobin, triglycerides, uric acid, LDL-C, and D-dimer. Finally, the predictive performance of FIB for moderate-to-severe decline in CRF was assessed using receiver operating characteristic (ROC) curves and the area under the curve (AUC). All tests were two-tailed, and a *P* value < 0.05 was considered statistically significant.

## 3 Results

### 3.1 Baseline characteristics according to cardiorespiratory fitness

As shown in Table 1, among the 698 participants, two groups were defined based on CRF: normal-to-mild decline (*n* = 292) and moderate-to-severe decline (*n* = 406). Compared with the normal-to-mild decline group, the moderate-to-severe decline group had a higher proportion of females, a greater prevalence of CHD, and higher levels of BMI, D-dimer, and FIB. In contrast, they had lower proportions of drinking and lower levels of peak O_2_ pulse, HDL-C, serum sodium, hemoglobin, and LVEF (all *P* < 0.05). No significant differences were observed between the two groups in age, smoking, family history of CHD, hypertension, hyperlipidemia, or diabetes (all *P* > 0.05). These findings indicate that multiple clinical markers, including FIB, are significantly partitioned according to the severity of CRF decline.

**Table 1:** Baseline characteristics stratified by cardiorespiratory fitness status.

Variables	Total population	Normal-to-mild decline	Moderate-to-severe decline	*P* value
N	698	292	406	
Age, years	59.00 (53.00, 65.00)	59.00 (53.00, 64.75)	59.00 (52.00, 66.00)	0.380
Gender, *n* (%)				0.041
Male	455 (65.2%)	203 (69.5%)	252 (62.1%)	
Female	243 (34.8%)	89 (30.5%)	154 (37.9%)	
BMI, kg/m^2^	25.33 (23.12, 27.63)	24.99 (22.82, 27.34)	25.71 (23.24, 27.76)	0.035
Smoking, *n* (%)	293 (42.0%)	124 (42.5%)	169 (41.6%)	0.824
Drinking, *n* (%)	193 (27.7%)	97 (33.2%)	96 (23.7%)	0.005
CHD family history, *n* (%)	104 (14.9%)	39 (13.4%)	65 (16.0%)	0.331
Hypertension, *n* (%)	488 (69.9%)	212 (72.6%)	276 (68.0%)	0.189
Hyperlipidemia, *n* (%)	232 (33.2%)	100 (34.3%)	132 (32.5%)	0.631
Diabetes, *n* (%)	222 (31.8%)	85 (29.1%)	137 (33.7%)	0.195
Stroke, *n* (%)	112 (16.0%)	49 (16.8%)	63 (15.5%)	0.654
CHD, *n* (%)	503 (72.1%)	194 (66.4%)	309 (76.1%)	0.005
PCI, *n* (%)	189 (27.1%)	76 (26.0%)	113 (27.8%)	0.597
Arrhythmia, *n* (%)	117 (16.8%)	52 (17.8%)	65 (16.0%)	0.530
Antihypertensive drugs, *n* (%)	465 (66.6%)	201 (68.8%)	264 (65.0%)	0.292
Antidiabetic drugs, *n* (%)	181 (25.9%)	68 (23.3%)	113 (27.8%)	0.177
Lipid-lowering drugs, *n* (%)	405 (58.0%)	157 (53.8%)	248 (61.1%)	0.053
Antiplatelet drugs, *n* (%)	378 (54.2%)	147 (50.3%)	231 (56.9%)	0.086
β-blocker, *n* (%)	227 (32.5%)	104 (35.6%)	123 (30.3%)	0.139
Peak O_2_ pulse, mL/beat	9.79 (7.81, 11.60)	10.45 (9.04, 12.44)	9.00 (7.22, 10.96)	<0.001
SBP, mmHg	130.00 (120.00, 142.00)	133.00 (120.25, 141.00)	130.00 (120.00, 143.00)	0.697
DBP, mmHg	77.00 (70.00, 85.00)	77.00 (70.00, 85.00)	77.00 (70.00, 84.00)	0.283
HR, bpm	70.00 (63.00, 78.00)	70.00 (63.00, 78.00)	70.00 (64.00, 78.00)	0.274
ALT, U/L	21.50 (16.00, 31.00)	21.00 (16.00, 30.00)	22.00 (16.00, 33.00)	0.076
AST, U/L	22.00 (18.00, 27.00)	21.00 (18.00, 26.00)	22.00 (18.00, 27.00)	0.170
Albumin, g/L	41.34 (39.30, 43.60)	41.34 (39.73, 43.08)	41.34 (38.90, 43.80)	0.421
Fasting glucose, mmol/L	5.30 (4.74, 6.14)	5.29 (4.71, 6.10)	5.30 (4.78, 6.17)	0.258
Total cholesterol, mmol/L	3.91 (3.29, 4.65)	3.97 (3.29, 4.65)	3.85 (3.27, 4.67)	0.706
Triglycerides, mmol/L	1.52 (1.10, 2.06)	1.48 (1.07, 1.91)	1.55 (1.13, 2.14)	0.204
HDL-C, mmol/L	1.02 (0.88, 1.15)	1.04 (0.89, 1.20)	0.99 (0.88, 1.12)	0.014
LDL-C, mmol/L	2.46 (1.94, 2.98)	2.42 (1.94, 2.94)	2.48 (1.92, 3.00)	0.352
eGFR, mL/min/1.73 m^2^	100.00 (93.00, 106.00)	100.00 (94.00, 106.00)	100.00 (93.00, 105.00)	0.095
Uric acid, ųmol/L	349.00 (286.50, 392.00)	349.04 (290.00, 394.75)	343.00 (280.00, 390.00)	0.208
Potassium, mmol/L	3.93 (3.72, 4.11)	3.93 (3.71, 4.15)	3.93 (3.73, 4.10)	0.669
Sodium, mmol/L	141.00 (140.00, 142.00)	141.00 (140.00, 143.00)	141.00 (140.00, 142.00)	0.011
Chloride, mmol/L	105.22 (103.60, 106.70)	105.22 (103.80, 106.68)	105.22 (103.50, 106.70)	0.775
Hemoglobin, g/L	142.51 (133.00, 152.25)	145.00 (135.00, 154.00)	142.00 (130.75, 152.00)	0.005
White blood cell count, ×10^9^/L	6.20 (5.10, 7.50)	6.10 (5.00, 7.30)	6.25 (5.20, 7.50)	0.169
Platelet count, ×10^9^/L	208.00 (170.00, 249.00)	209.00 (169.00, 249.75)	208.00 (172.00, 248.25)	0.891
D-dimer, mg/L	0.35 (0.19, 15.25)	0.30 (0.18, 0.76)	0.38 (0.20, 24.03)	0.006
LVEF, %	62.00 (58.00, 66.00)	63.00 (59.00, 66.00)	62.00 (57.00, 65.00)	0.012
FIB, g/L	2.78 (2.44, 3.23)	2.69 (2.35, 3.11)	2.86 (2.51, 3.29)	<0.001

Categorical variables are presented as *n (%)* and were compared using the *χ*^2^ test. Continuous variables are presented as median [interquartile range (IQR)] and were compared using the nonparametric Mann–Whitney *U*-test. Peak O_2_ pulse was calculated as VO_2_ peak divided by peak heart rate (VO_2_ peak/HR peak). BMI, body mass index; CHD, coronary heart disease; PCI, percutaneous coronary intervention; SBP, systolic blood pressure; DBP, diastolic blood pressure; HR, heart rate; ALT, alanine aminotransferase; AST, aspartate aminotransferase; HDL-C, high-density lipoprotein cholesterol; LDL-C, low-density lipoprotein cholesterol; eGFR, estimated glomerular filtration rate; LVEF, left ventricular ejection fraction; FIB, fibrinogen.

### 3.2 Characteristics according to fibrinogen tertiles

As shown in Table 2, participants were categorized into three FIB tertiles: T1 (*n* = 238), T2 (*n* = 228), and T3 (*n* = 232). Significant differences were observed among the three groups for several variables, including gender, peak O_2_ pulse, HR, albumin, TC, eGFR, serum sodium and chloride, hemoglobin, WBC, platelet count, D-dimer, and CRF (all *P* < 0.05). Notably, the proportion of participants with moderate-to-severe CRF decline increased progressively across FIB tertiles (T1: 49.6%, T2: 60.1%, T3: 65.1%; *P* = 0.002). Simultaneously, peak O_2_ pulse decreased across increasing FIB tertiles. Overall, higher FIB levels were associated with an unfavorable cardiopulmonary profile, characterized by a progressively higher prevalence of moderate-to-severe CRF decline and a concomitant reduction in peak O_2_ pulse across tertiles.

**Table 2:** Characteristics of variables grouped by fibrinogen tertiles.

Variables	T1	T2	T3	*P* value
N	238	228	232	
Age, years	58.00 (52.00, 65.00)	59.00 (53.00, 65.00)	60.00 (53.00, 66.00)	0.287
Gender, *n* (%)				<0.001
Male	179 (75.2%)	140 (61.4%)	136 (58.6%)	
Female	59 (24.8%)	88 (38.6%)	96 (41.4%)	
BMI, kg/m^2^	24.91 (22.77, 27.05)	25.59 (23.14, 27.68)	25.44 (23.46, 27.78)	0.071
Smoking, *n* (%)	109 (45.8%)	92 (40.4%)	92 (39.7%)	0.335
Drinking, *n* (%)	73 (30.7%)	61 (26.8%)	59 (25.4%)	0.417
CHD family history, *n* (%)	42 (17.6%)	32 (14.0%)	30 (12.9%)	0.323
Hypertension, *n* (%)	163 (68.5%)	160 (70.2%)	165 (71.1%)	0.819
Hyperlipidemia, *n* (%)	78 (32.8%)	78 (34.2%)	76 (32.8%)	0.930
Diabetes, *n* (%)	79 (33.2%)	73 (32.0%)	70 (30.2%)	0.778
Stroke, *n* (%)	35 (14.7%)	38 (16.7%)	39 (16.8%)	0.785
CHD, *n* (%)	169 (71.0%)	159 (69.7%)	175 (75.4%)	0.358
PCI, *n* (%)	70 (29.4%)	56 (24.6%)	63 (27.2%)	0.499
Arrhythmia, *n* (%)	43 (18.1%)	37 (16.2%)	37 (15.9%)	0.800
Antihypertensive drugs, *n* (%)	157 (66.0%)	152 (66.7%)	156 (67.2%)	0.958
Antidiabetic drugs, *n* (%)	63 (26.5%)	56 (24.6%)	62 (26.7%)	0.846
Lipid-lowering drugs, *n* (%)	140 (58.8%)	134 (58.8%)	131 (56.5%)	0.841
Antiplatelet drugs, *n* (%)	136 (57.1%）	125 (54.8%）	117 (50.4%)	0.334
β-blocker, *n* (%)	82 (34.5%)	71 (31.1%)	74 (31.9%)	0.725
peak O_2_ pulse, mL/beat	10.10 (8.65, 12.11)	9.89 (7.94, 11.58)	8.93 (7.20, 11.29)	<0.001
SBP, mmHg	131.00 (120.00, 140.00)	130.50 (120.00, 142.00)	130.00 (121.00, 144.00)	0.765
DBP, mmHg	77.00 (70.75, 85.00)	78.00 (69.00, 85.00)	77.00 (70.00, 84.00)	0.578
HR, bpm	69.00 (62.00, 77.00)	70.00 (64.00, 78.00)	72.00 (64.00, 79.00)	0.017
ALT, U/L	23.00 (17.00, 32.00)	21.00 (16.00, 32.75)	21.00 (16.00, 30.00)	0.362
AST, U/L	22.00 (18.00, 26.50)	22.00 (18.00, 26.50)	22.00 (18.00, 27.00)	0.544
Albumin, g/L	41.34 (39.43, 43.15)	41.90 (39.70, 44.00)	41.10 (38.70, 43.48)	0.007
Fasting glucose, mmol/L	5.17 (4.71, 5.88)	5.40 (4.79, 6.21)	5.34 (4.77, 6.28)	0.193
Total cholesterol, mmol/L	3.81 (3.23, 5.56)	3.86 (3.22, 5.53)	4.06 (3.35, 4.85)	0.043
Triglycerides, mmol/L	1.52 (1.10, 1.98)	1.48 (1.12, 2.13)	1.56 (1.09, 2.08)	0.812
HDL-C, mmol/L	1.01 (0.90, 1.15)	1.01 (0.88, 1.16)	1.03 (0.86, 1.14)	0.928
LDL-C, mmol/L	2.35 (1.88, 2.93)	2.45 (1.94, 2.86)	2.52 (2.04, 3.17)	0.051
eGFR, mL/min/1.73 m^2^	102.00 (96.00, 107.00)	101.00 (94.00, 105.75)	98.00 (87.25, 104.75)	<0.001
Uric acid, ųmol/L	349.04 (283.75, 393.25)	341.00 (284.00, 389.50)	349.04 (290.00, 400.00)	0.547
Potassium, mmol/L	3.92 (3.71, 4.09)	3.93 (3.72, 4.10)	3.93 (3.73, 4.14)	0.243
Sodium, mmol/L	141.00 (140.00, 143.00)	141.00 (140.00, 142.00)	141.00 (140.00, 142.00)	0.049
Chloride, mmol/L	105.40 (104.33, 107.10)	105.10 (103.23, 106.20)	105.21 (103.20, 106.40)	0.001
Hemoglobin, g/L	145.50 (135.00, 154.00)	142.00 (133.00, 153.00)	140.00 (129.00, 151.00)	0.004
White blood cell count, ×10^9^/L	5.90 (4.88, 7.03)	5.90 (5.03, 7.40)	6.70 (5.70, 7.90)	<0.001
Platelet count, ×10^9^/L	198.00 (163.00, 241.00)	199.50 (167.00, 240.75)	222.00 (186.25, 261.75)	<0.001
D-dimer, mg/L	0.24 (0.16, 0.56)	0.35 (0.19, 24.03)	0.42 (0.27, 34.25)	<0.001
LVEF, %	63.00 (59.00, 66.00)	62.00 (58.00, 65.00)	62.00 (57.00, 66.00)	0.196
Cardiorespiratory fitness, *n* (%)				0.002
Normal-to-mild decline	120 (50.4%)	91 (39.9%)	81 (34.9%)	
Moderate-to-severe decline	118 (49.6%)	137 (60.1%)	151 (65.1%)	

Categorical variables are presented as *n (%)* and were compared using the *χ*² test. Continuous variables are presented as median (IQR) and were compared using the nonparametric Kruskal–Wallis H test. Fibrinogen tertiles were defined as T1 (≤2.58 g/L), T2 (2.59–3.07 g/L), and T3 (>3.07 g/L). Peak O_2_ pulse was calculated as VO_2_ peak divided by peak heart rate (VO_2_ peak/HR peak). BMI, body mass index; CHD, coronary heart disease; PCI, percutaneous coronary intervention; SBP, systolic blood pressure; DBP, diastolic blood pressure; HR, heart rate; ALT, alanine aminotransferase; AST, aspartate aminotransferase; HDL-C, high-density lipoprotein cholesterol; LDL-C, low-density lipoprotein cholesterol; eGFR, estimated glomerular filtration rate; LVEF, left ventricular ejection fraction.

### 3.3 Univariate logistic regression analysis

As presented in Table 3, univariate logistic regression indicated that gender, BMI, drinking, CHD, fasting glucose, HDL-C, eGFR, serum sodium, hemoglobin, LVEF, and FIB were significantly associated with the risk of moderate-to-severe CRF decline (all *P* < 0.05). Specifically, each 1 g/L increase in FIB was associated with a 47.6% increase risk of moderate-to-severe CRF decline (OR = 1.476, *P* < 0.001). Compared with T1, the risk of moderate-to-severe CRF decline was 1.531-fold higher in T2 (OR = 1.531, *P* = 0.023) and 1.896-fold higher in T3 (OR = 1.896, *P* < 0.001). Taken together, higher FIB levels were significantly associated with an increased risk of moderate-to-severe CRF decline.

**Table 3:** Univariable logistic regression for moderate-to-severe cardiorespiratory fitness decline.

Variables	OR	95% CI	*P* value
Age	1.007	0.992–1.023	0.350
Male	0.717	0.521–0.988	0.042
BMI	1.055	1.008–1.105	0.023
Smoking	0.966	0.712–1.310	0.824
Drinking	0.623	0.446–0.870	0.005
CHD family history	1.237	0.805–1.899	0.332
Hypertension	0.801	0.575–1.116	0.189
Hyperlipidemia	0.925	0.673–1.272	0.631
Diabetes	1.240	0.896–1.718	0.195
Stroke	0.911	0.606–1.370	0.654
CHD	1.609	1.153–2.246	0.005
PCI	1.096	0.780–1.540	0.597
Arrhythmia	0.880	0.590–1.313	0.531
Antihypertensive drugs	0.842	0.611–1.160	0.292
Antidiabetic drugs	1.270	0.898–1.798	0.177
Lipid-lowering drugs	1.350	0.995–1.830	0.054
Antiplatelet drugs	1.302	0.963–1.761	0.087
β-blocker use	1.273	0.925–1.752	0.139
SBP	1.004	0.993–1.016	0.448
DBP	0.984	0.968–1.001	0.067
HR	1.009	0.996–1.022	0.169
ALT	1.003	0.996–1.011	0.361
AST	1.009	1.000–1.019	0.054
Albumin	0.979	0.936–1.024	0.350
Fasting glucose	1.116	1.025–1.214	0.011
Total cholesterol	1.007	0.875–1.158	0.925
Triglycerides	0.972	0.866–1.090	0.625
HDL-C	0.384	0.203–0.728	0.003
LDL-C	1.143	0.943–1.385	0.174
eGFR	0.985	0.973–0.997	0.015
Uric acid	0.999	0.998–1.001	0.407
Potassium	0.914	0.586–1.424	0.691
Sodium	0.899	0.837–0.967	0.004
Chloride	0.985	0.935–1.038	0.580
Hemoglobin	0.985	0.975–0.995	0.003
White blood cell count	1.070	0.989–1.158	0.090
Platelet count	1.000	0.998–1.003	0.759
D-dimer	1.002	1.000–1.005	0.080
LVEF	0.974	0.956–0.993	0.007
FIB (continuous variable)	1.476	1.189–1.834	<0.001
FIB (categorical variable)			
T1	Ref		
T2	1.531	1.061–2.210	0.023
T3	1.896	1.309–2.747	<0.001

Univariable logistic regression analyses were performed to evaluate the association between each clinical variable and moderate-to-severe CRF decline. BMI, body mass index; CHD, coronary heart disease; PCI, percutaneous coronary intervention; SBP, systolic blood pressure; DBP, diastolic blood pressure; HR, heart rate; ALT, alanine aminotransferase; AST, aspartate aminotransferase; HDL-C, high-density lipoprotein cholesterol; LDL-C, low-density lipoprotein cholesterol; eGFR, estimated glomerular filtration rate; LVEF, left ventricular ejection fraction; FIB, fibrinogen.

### 3.4 Association between fibrinogen and moderate-to-severe CRF decline

**Figure 2:**
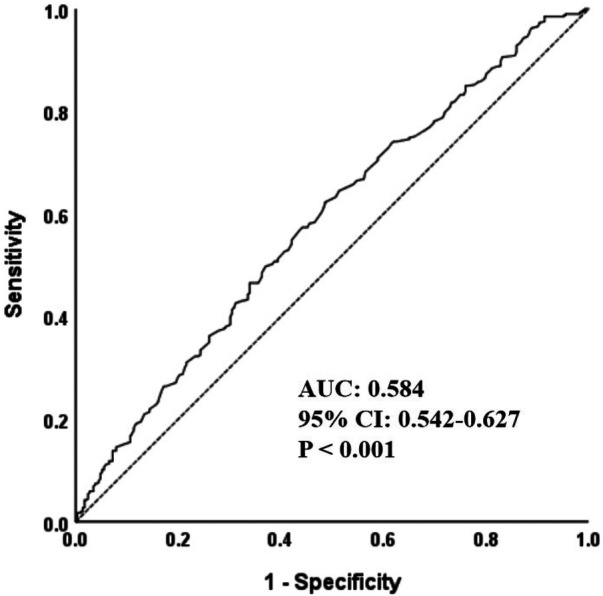
ROC curve for fibrinogen in predicting moderate-to-severe cardiorespiratory fitness decline. ROC, receiver operating characteristic curve; AUC, area under the curve.

As shown in Table 4, multivariable logistic regression confirmed that this association remained robust after adjusting for confounders. In Model 2 (adjusted for gender, drinking, and CHD), each 1 g/L increase in FIB was associated with a 43.0% higher risk of moderate-to-severe CRF decline (OR = 1.430, *P* = 0.001). Compared with T1, the risk in T2 and T3 was 1.495-fold (OR = 1.495, *P* = 0.035) and 1.785-fold higher (OR = 1.785, *P* = 0.003), respectively. In the fully adjusted Model 3(further controlling for fasting glucose, HDL-C, eGFR, hemoglobin, and LVEF), higher FIB remained significantly associated with moderate-to-severe CRF decline. Specifically, each 1 g/L increase in FIB increased the risk by 31.4% (OR = 1.314, 95% CI: 1.051–1.643, *P* = 0.016), and participants in T3 had a 1.620-fold higher risk than those in T1 (OR = 1.620, 95% CI: 1.101–2.383, *P* = 0.014). As shown in Figure 2, ROC curve analysis further supported the predictive value of FIB for moderate-to-severe CRF decline (AUC = 0.584, *P* < 0.001). Collectively, these findings demonstrate that FIB is an independent risk factor for CRF decline.

**Table 4:** Multivariable logistic regression analysis.

Variables	Model 1		Model 2		Model 3	
	OR (95% CI)	*P* value	OR (95% CI)	*P* value	OR (95% CI)	*P* value
FIB (continuous variable)	1.476 (1.189, 1.834)	<0.001	1.430 (1.151, 1.777)	0.001	1.314 (1.051, 1.643)	0.016
FIB (categorical variable)						
T1	Ref					
T2	1.531 (1.061, 2.210)	0.023	1.495 (1.028, 2.173)	0.035	1.439 (0.983, 2.106)	0.061
T3	1.896 (1.309, 2.747)	<0.001	1.785 (1.222, 2.607)	0.003	1.620 (1.101, 2.383)	0.014

Model 1: unadjusted; Model 2: adjusted for gender, drinking, and CHD; Model 3: adjusted for gender, drinking, CHD, fasting glucose, HDL-C, eGFR, hemoglobin, and LVEF. FIB, fibrinogen; CHD, coronary heart disease; HDL-C, high-density lipoprotein cholesterol; eGFR, estimated glomerular filtration rate; LVEF, left ventricular ejection fraction.

### 3.5 Subgroup analysis of fibrinogen and moderate-to-severe CRF decline

As shown in Table 5, subgroup analyses demonstrated that the positive association between FIB and the risk of moderate-to-severe CRF decline remained consistent across various clinical strata.

**Table 5:** Subgroup analysis of the association between fibrinogen and moderate-to-severe cardiorespiratory fitness decline.

Subgroups	FIB		FIB (T2 vs. T1)		FIB (T3 vs. T1)	
	OR (95% CI)	*P* value	OR (95% CI)	*P* value	OR (95% CI)	*P* value
Age						
<60 years	1.287 (0.935, 1.773)	0.122	1.091 (0.646, 1.844)	0.744	1.366 (0.783, 2.383)	0.272
≥60 years	1.397 (1.024, 1.907)	0.035	2.091 (1.186, 3.687)	0.011	1.910 (1.085, 3.363)	0.025
Gender						
Male	1.406 (1.065, 1.856)	0.016	1.236 (0.773, 1.975)	0.376	1.510 (0.931, 2.447)	0.095
Female	1.002 (0.658, 1.525)	0.994	1.670 (0.836, 3.336)	0.147	1.638 (0.816, 3.288)	0.165
BMI						
<24 kg/m^2^	1.470 (1.003, 2.157)	0.048	1.733 (0.910, 3.300)	0.094	2.431 (1.264, 4.676)	0.008
≥24 kg/m^2^	1.294 (0.984, 1.702)	0.065	1.302 (0.806, 2.102)	0.281	1.299 (0.796, 2.118)	0.295
Smoking						
Yes	1.272 (0.835, 1.936)	0.262	1.273 (0.691, 2.344)	0.439	1.153 (0.627, 2.120)	0.647
No	1.427 (1.096, 1.858)	0.008	1.544 (0.950, 2.509)	0.080	2.281 (1.391, 3.743)	0.001
Hypertension						
Yes	1.185 (0.911, 1.541)	0.207	1.159 (0.734, 1.829)	0.526	1.463 (0.915, 2.340)	0.112
No	1.655 (1.065, 2.572)	0.025	2.331 (1.154, 4.709)	0.018	1.926 (0.964, 3.846)	0.063
Hyperlipidemia						
Yes	1.170 (0.797, 1.716)	0.423	1.404 (0.718, 2.746)	0.321	1.856 (0.921, 3.741)	0.084
No	1.546 (1.183, 2.021)	0.001	1.434 (0.897, 2.291)	0.132	1.393 (0.862, 2.250)	0.176
Diabetes						
Yes	0.825 (0.544, 1.252)	0.367	0.665 (0.332, 1.332)	0.250	0.984 (0.456, 2.126)	0.968
No	1.619 (1.219, 2.151)	<0.001	2.143 (1.359, 3.381)	0.001	2.063 (1.317, 3.231)	0.002
CHD						
Yes	1.395 (1.072, 1.815)	0.013	1.481 (0.944, 2.322)	0.087	1.776 (1.129, 2.794)	0.013
No	1.175 (0.741, 1.863)	0.492	1.417 (0.682, 2.942)	0.350	1.313 (0.600, 2.873)	0.495

Subgroup analysis adjusted for gender, drinking, CHD, fasting glucose, HDL-C, eGFR, hemoglobin, and LVEF. FIB, fibrinogen; BMI, body mass index; CHD, coronary heart disease; HDL-C, high-density lipoprotein cholesterol; eGFR, estimated glomerular filtration rate; LVEF, left ventricular ejection fraction.

Demographic subgroups: In participants aged ≥60 years, each 1 g/L increase in FIB was associated with a 39.7% higher risk of moderate-to-severe CRF decline (OR = 1.397, *P* = 0.035), and the risk in T2 and T3 was 2.091-fold (OR = 2.091, *P* = 0.011) and 1.910-fold higher (OR = 1.910, *P* = 0.025), respectively. Similarly, in men, each 1 g/L increase in FIB increased the risk by 40.6% (OR = 1.406, *P* = 0.016).

Anthropometric subgroup: In those with a BMI <24 kg/m^2^, each 1 g/L increase in FIB increased the risk by 47.0% (OR = 1.470, *P* = 0.048), and the risk in T3 was 2.431-fold higher than T1 (OR = 2.431, *P* = 0.008). Furthermore, among non-smokers, the risk increased by 42.7% per unit FIB increase (OR = 1.427, *P* = 0.008), and the risk in T3 was 2.281-fold higher than T1 (OR = 2.281, *P* = 0.001).

Comorbidity Subgroups: In participants without hypertension, each 1 g/L increase in FIB increased the risk by 65.5% (OR = 1.655, *P* = 0.025), and the risk in T2 was 2.331-fold higher than T1 (OR = 2.331, *P* = 0.018). In participants without hyperlipidemia, each 1 g/L increase in FIB increased the risk by 54.6% (OR = 1.546, *P* = 0.001). In participants without diabetes, each 1 g/L increase in FIB increased the risk by 61.9% (OR = 1.619, *P* < 0.001), and the risk in T2 and T3 was 2.143-fold (OR = 2.143, *P* = 0.001) and 2.063-fold higher (OR = 2.063, *P* = 0.002), respectively, compared with T1. Furthermore, in patients with CHD, each 1 g/L increase in FIB was associated with a 39.5% higher risk of moderate-to-severe CRF decline (OR = 1.395, *P* = 0.013), and the risk in T3 was 1.776-fold higher than T1 (OR = 1.776, *P* = 0.013).

Overall, these findings indicate that elevated FIB levels are consistently associated with an increased risk of moderate-to-severe CRF decline, irrespective of baseline demographic characteristics、anthropometric measurements or comorbidities.

### 3.6 Sensitivity analysis

As shown in Table 6, to verify the robustness of our findings, multivariable logistic regression analyses were repeated in the 388 participants who reached maximal exercise effort (RER ≥1.10). In the unadjusted Model 1, each 1-unit increase in FIB was associated with a 172.4% higher risk of moderate-to-severe decline in CRF (OR = 2.724, *P* < 0.001). Compared with the T1 group, the risks of moderate-to-severe decline in the T2 and T3 groups were 1.969-fold (OR = 1.969, *P* = 0.009) and 3.842-fold (OR = 3.842, *P* < 0.001), respectively. In Model 2 (adjusted for gender, drinking, and CHD), each 1-unit increase in FIB was associated with a 157.3% higher risk of moderate-to-severe decline in CRF (OR = 2.573, 9, *P* < 0.001). Compared with T1, the risks in T2 and T3 were 1.904-fold (OR = 1.904, *P* = 0.016) and 3.518-fold (OR = 3.518, *P* < 0.001), respectively. In the fully adjusted Model 3 (additionally controlled for fasting glucose, HDL-C, eGFR, hemoglobin, and LVEF), higher FIB levels remained significantly associated with the risk of moderate-to-severe decline in CRF. Specifically, each 1-unit increase in FIB was associated with a 157.3% higher risk (OR = 2.573, *P* < 0.001), and the risks in T2 and T3 were 1.817-fold (OR = 1.817, *P* = 0.027) and 3.306-fold (OR = 3.306, *P* < 0.001), respectively, compared with T1. This sensitivity analysis further confirms that the association between elevated FIB and CRF decline is particularly pronounced in individuals who perform at their maximal physiologic capacity.

**Table 6:** Sensitivity analyses excluding participants who did not achieve maximal effort.

Variables	Model 1		Model 2		Model 3	
	OR (95% CI)	*P* value	OR (95% CI)	*P* value	OR (95% CI)	*P* value
FIB (continuous variable)	2.724 (1.922, 3.861)	<0.001	2.573 (1.815, 3.648)	<0.001	2.573 (1.815, 3.648)	<0.001
FIB (categorical variable)						
T1	Ref					
T2	1.969 (1.183, 3.279)	0.009	1.904 (1.130, 3.210)	0.016	1.817 (1.072, 3.080)	0.027
T3	3.842 (2.282, 6.468)	<0.001	3.518 (2.068, 5.985)	<0.001	3.306 (1.934, 5.652)	<0.001

In this sensitivity analysis, participants with RER <1.1 were considered to have not achieved maximal effort and were excluded.

Model 1: unadjusted; Model 2: adjusted for gender, drinking, and CHD; Model 3: adjusted for gender, drinking, CHD, fasting glucose, HDL-C, eGFR, hemoglobin, and LVEF.

RER, respiratory exchange ratio; FIB, fibrinogen; CHD, coronary heart disease; HDL-C, high-density lipoprotein cholesterol; eGFR, estimated glomerular filtration rate; LVEF, left ventricular ejection fraction.

### 3.7 Association between fibrinogen and peak O_2_ pulse

As shown in Table 7, multivariable linear regression analyses demonstrated an inverse association between FIB levels and peak O_2_ pulse. In the unadjusted model, each 1 g/L increase in FIB was associated with a lower peak O_2_ pulse (*β* = −0.558, *P* < 0.001). When analyzed by tertiles, participants in T3 had a significantly lower peak O_2_ pulse than those in T1 (*β* = −1.003, *P* < 0.001). In Model 2, after adjustment for gender, smoking, drinking, and β-blocker use, the inverse association remained significant (per 1 g/L increase: *β* = −0.401, *P* < 0.001), and T3 was still associated with a lower peak O_2_ pulse compared with T1 (*β* = −0.472, *P* = 0.027). In the fully adjusted Model 3 (adjusting for gender, smoking, drinking, β-blocker use, age, BMI, DBP, ALT, platelet count, hemoglobin, triglycerides, uric acid, LDL-C, and D-dimer), the association persisted. Specifically, each 1 g/L increase in FIB was associated with a 0.357 mL/beat lower peak O_2_ pulse (*β* = −0.357, *P* < 0.001), and participants in T3 had a significantly lower peak O_2_ pulse than those in T1 (*β* = −0.503, *P* = 0.012). These results provide strong evidence that elevated FIB is independently linked to impaired stroke volume or peripheral oxygen extraction efficiency during maximal exertion, as reflected by the reduction in peak O_2_ pulse.

**Table 7:** Association between fibrinogen and peak O_2_ pulse.

Variables	Model 1		Model 2		Model 3	
	β (95% CI)	*P* value	β (95% CI)	*P* value	β (95% CI)	*P* value
FIB (continuous variable)	−0.558 (−0.816, −0.300)	<0.001	−0.401 (−0.616, −0.185)	<0.001	−0.357 (−0.564, −0.151)	<0.001
FIB (categorical variable)						
T1	Ref					
T2	−0.459 (−0.956, 0.038)	0.070	−0.014 (−0.432, 0.404)	0.947	−0.089 (−0.475, 0.297)	0.652
T3	−1.003 (−1.498, −0.508)	<0.001	−0.472 (−0.889, −0.054)	0.027	−0.503 (−0.897, −0.109)	0.012

Outcome: peak O_2_ pulse (mL/beat). Model 1: unadjusted; Model 2: adjusted for gender, smoking, drinking and β-blocker use; Model 3: adjusted for gender, smoking, drinking, β-blocker use, age, BMI, DBP, ALT, platelet count, hemoglobin, triglycerides, uric acid, LDL-C, and D-dimer. FIB, fibrinogen; BMI, body mass index; DBP, diastolic blood pressure; ALT, alanine aminotransferase; LDL-C, low-density lipoprotein cholesterol.

## 4 Discussion

In this real-world, single-center retrospective study, multivariable logistic regression analysis demonstrated that elevated FIB levels were significantly associated with an increased risk of moderate-to-severe decline. This association remained robust across subgroup analyses stratified by common clinical variables. During CPET, insufficient effort often leads to an underestimation of VO_2_ peak and greater measurement variability (34). Therefore, we performed a sensitivity analysis restricted to participants who achieved maximal effort during CPET. The results showed that the association between FIB and moderate-to-severe CRF decline persisted and exhibited a stronger effect size, further supporting the true relationship between FIB and CRF. Moreover, in complementary analyses, higher FIB levels were independently associated with a lower peak O_2_ pulse, providing additional support for a link between elevated FIB and impaired cardiovascular response during exercise. Additionally, ROC curve analysis indicated that FIB had potential predictive value for moderate-to-severe CRF decline in clinical populations undergoing CPET, suggesting its utility as a readily accessible biomarker for functional risk stratification; however, these findings warrant further validation in larger and prospective studies.

Extensive evidence has established the clinical relevance of FIB in CVD, with elevated levels consistently associated with greater disease burden and worse outcomes. A large individual-participant meta-analysis demonstrated that higher plasma FIB levels were independently associated with increased risks of CHD, ischemic stroke, and vascular mortality (42). In patients with established CHD, cross-sectional and cohort studies have further linked elevated FIB to more extensive or severe coronary lesions and to a higher risk of adverse events after PCI (43). A retrospective cohort study involving 43,367 Chinese patients with CHD demonstrated that elevated FIB levels were independently associated with a significantly increased risk of all-cause mortality, and that combining FIB with lipoprotein(a) further improved prognostic accuracy (44). Moreover, recent studies suggest that FIB and its derived indices (e.g., FIB-to-albumin ratio) can provide risk stratification in patients with heart failure and critical illness, further supporting FIB as an accessible cardiovascular risk biomarker (45). Moreover, increasing evidence suggests that dysregulation of coagulation and fibrinolytic systems may contribute to CRF decline. For example, in athletes, elevated D-dimer levels were associated with a lower percentage of maximal power achieved during CPET, suggesting reduced exercise efficiency or limited cardiovascular adaptation (11). In pediatric cohorts at high risk for obesity and diabetes, VO_2_ max was inversely correlated with FIB, and FIB demonstrated predictive value for VO_2_ max (12). Similarly, in a community adult cohort, D-dimer levels were positively correlated with body fat percentage, plasminogen activator inhibitor-1 was negatively correlated with VO_2_ peak, and thrombomodulin was positively correlated with VO_2_ peak, indicating that activation of the coagulation system may reflect reduced cardiopulmonary metabolic reserve (13). Collectively, these findings suggest that elevated coagulation markers may not only reflect chronic inflammation and metabolic dysregulation but may also contribute to reduced exercise tolerance and CRF through multiple mechanisms. Nevertheless, prior research has largely focused on athletes, community populations, or specific pediatric subgroups and differences in outcome definitions and effort control have contributed to considerable heterogeneity across studies. Therefore, the relationship between FIB and CRF requires further clarification. In this context, we evaluated FIB in a real-world clinical cohort including both patients with established CVD and individuals presenting with cardiac-related symptoms but without confirmed structural heart disease, thus better reflecting routine clinical practice. Moreover, we undergo CPET—the “gold standard” for objective CRF assessment. We found that elevated FIB levels were significantly associated with moderate-to-severe CRF decline, and this association was consistent across major clinical subgroups. Moreover, we performed a sensitivity analysis restricted to participants who achieved maximal effort to reduce underestimation and measurement variability of VO_2_ peak due to submaximal effort, thereby providing more robust evidence that is less influenced by subjective motivation. Furthermore, the inverse association identified between FIB and peak O_2_ pulse offers critical mechanistic granularity, suggesting that elevated FIB may compromise the integrated efficiency of central stroke volume and peripheral oxygen extraction. Overall, our findings provide more direct, quantifiable, and clinically interpretable evidence supporting the association between FIB and CRF decline, and they lay a foundation for future prospective validation and functional risk stratification based on routinely available laboratory measures. Nevertheless, the underlying biological mechanisms by which FIB contributes to CRF decline remain incompletely understood and warrant further investigation.

From a mechanistic perspective, FIB is not only a key substrate for fibrin formation in the coagulation cascade but also a typical acute-phase reactant. The association between elevated FIB and CRF decline may be driven primarily by three interconnected mechanistic axes: systemic inflammation, oxidative stress, and microcirculatory dysfunction. These pathways may interact and reinforce one another, ultimately restricting oxygen delivery and utilization during exercise and thereby contributing to reduced CRF. First, the systemic inflammation axis. Elevated FIB often indicates a background of sustained inflammatory activation. In inflammatory or tissue-injury settings, FIB may act not only as a marker of inflammatory burden but also as a functional mediator: it can be converted and deposited as fibrin (and its degradation products), generating pro-inflammatory matrix cues that promote leukocyte adhesion and migration and enhance phagocytosis, inflammation-related gene transcription, and the release of cytokines and chemokines. These effects can amplify inflammation and aggravate endothelial dysfunction and dysregulated vascular control (20, 22, 46). Inflammation-related endothelial activation and reduced vascular reactivity can impair exercise-induced blood flow redistribution and peripheral perfusion regulation (47, 48). In addition, inflammatory states may reduce skeletal muscle oxygen uptake and utilization, thereby promoting CRF decline (49, 50). Importantly, inflammation frequently coexists with a prothrombotic tendency, providing a basis for increased micro-thrombotic burden and impaired microcirculatory perfusion, which further limits the matching of oxygen delivery and utilization during exercise (51). Second, the oxidative stress axis. Notably, recent studies suggest that oxidative stress can modify the molecular structure of FIB, resulting in denser and more fibrinolysis-resistant fibrin networks. This “hypofibrinolytic” thrombotic phenotype not only increases thrombotic risk but may also cause more persistent perfusion impairment and chronic ischemia–hypoxia at the microvascular level (52, 53). As oxygen consumption approaches its peak during exercise, these dense micro-thrombotic networks can induce regional ischemia and hypoxia, ultimately manifesting as a decline in CRF. Third, the microcirculatory dysfunction axis. FIB is a major determinant of plasma viscosity; its elevation increases blood viscosity, promotes erythrocyte aggregation, and impairs microcirculatory perfusion, thereby reducing tissue oxygen delivery and utilization (54). Moreover, elevated FIB can facilitate fibrin network formation and enhance platelet aggregation, making thrombosis or microembolization more likely in the coronary and pulmonary circulations. This may lead to regional hypoperfusion and ischemia–hypoxia, further compromising cardiopulmonary reserve and exercise tolerance (55, 56). It is worth emphasizing that such perfusion limitation may occur not only at the microvascular level but may also manifest clinically as more severe cardiopulmonary vascular disease, thereby further amplifying the risk of CRF decline (57, 58). Based on the three mechanistic axes discussed above, the independent inverse association observed between FIB and peak O_2_ pulse in our study can be explained within a coherent physiological framework. Peak O_2_ pulse is commonly regarded as a practical approximation derived from the Fick principle: O_2_ pulse ≈ SV × C (a—v)O_2_, where stroke volume (SV) reflects the central pumping response and the arteriovenous oxygen difference C (a—v) O_2_ reflects peripheral oxygen extraction and utilization, particularly in skeletal muscle (59, 60). First, as a major determinant of plasma viscosity, elevated FIB may increase blood viscosity and promote erythrocyte aggregation, thereby reducing capillary perfusion efficiency and the effective exchange surface area, which could blunt the exercise-induced rise in C (a—v) O_2_. Meanwhile, increased peripheral resistance and impaired perfusion may raise cardiac afterload and, at maximal exercise, limit further augmentation of SV (61–64). Second, inflammation-related endothelial activation and impaired vasodilatory responsiveness may attenuate exercise-induced blood flow redistribution and peripheral perfusion regulation, reducing central–peripheral coupling efficiency; in addition, mitochondrial dysfunction in the context of chronic inflammation may further compromise muscular oxygen utilization, thereby constraining increases in C (a—v) O_2_ (65–67). Finally, oxidative modifications may promote the formation of denser, fibrinolysis-resistant fibrin networks and increase microthrombotic burden. This leads to persistent microvascular perfusion impairment and localized ischemia–hypoxia, disrupting the oxygen transport cascade (68, 69). Taken together, elevated FIB may reduce peak O_2_ pulse by limiting exercise-related increases in SV and/or C (a—v) O_2_, providing an integrated Fick principle–based explanation for our observation that higher FIB is associated with impaired cardiovascular responses during exercise. This framework also suggests that future studies integrating noninvasive cardiac output assessment and peripheral muscle oxygenation measurements may help quantify the relative contributions of central and peripheral components. Overall, the mechanisms outlined above provide a biologically plausible framework in which elevated FIB may both reflect and contribute to a self-amplifying thrombo-inflammatory milieu, together with increased oxidative stress and microcirculatory dysfunction, thereby limiting oxygen delivery and utilization during exercise and leading to CRF decline. Moreover, the inverse association between FIB and peak O_2_ pulse is consistent with this framework, suggesting that FIB-related hemorheological disturbances, endothelial dysfunction, and microcirculatory abnormalities may weaken central–peripheral coupling during exercise and manifest as an impaired cardiovascular response to exertion. Nevertheless, these inferences do not establish causality, and additional, as-yet-unidentified pathways may be involved. Future studies are warranted to identify potential mediators, quantify the relative contributions of these mechanisms, and validate causal pathways in prospective and mechanistic investigations.

Despite these positive findings, several limitations should be acknowledged. First, as a single-center retrospective study of hospitalized patients undergoing CPET, selection bias may limit the generalizability of our results, particularly to different regions, ethnicities, or community populations. Future multicenter, large-scale, and prospective studies are warranted. Second, due to the observational nature of the study, genetic or randomized evidence is lacking, precluding causal inference between FIB and CRF decline. Third, unmeasured confounders, including genetic background, occupational activity, diet, environmental factors, and socioeconomic status, may have influenced the results. Fourth, FIB was measured only once, without recording the timing relative to acute events or inflammatory episodes, limiting assessment of temporal dynamics between FIB and CRF. Finally, although all participants underwent standardized CPET, only CRF and peak O_2_ pulse were analyzed, and other CPET parameters were not systematically evaluated. Future studies should collect additional relevant metrics to comprehensively examine the relationship between FIB and CPET-derived clinical parameters.

## 5 Conclusion

This study demonstrated a significant association between higher serum FIB levels and moderate-to-severe CRF decline in patients undergoing CPET. In additional analyses, higher FIB was also inversely associated with peak O_2_ pulse, suggesting an attenuated cardiovascular response during exercise. These findings extend current evidence regarding the clinical relevance of FIB in relation to functional impairment and suggest its potential value as a readily available marker for identifying individuals at risk of cardiorespiratory dysfunction. However, given the single-center, retrospective design and the inherent limitations of this study, further prospective, multicenter studies with larger sample sizes are warranted. Future research integrating mechanistic investigations, genetic approaches, multi-marker panels, and artificial intelligence–based predictive models is needed to confirm these findings, clarify underlying mechanisms, and assess the incremental clinical utility of FIB across different populations and clinical settings.

## Data Availability

The raw data supporting the conclusions of this article will be made available by the authors, without undue reservation.
